# Reconceptualising Work and Employment in Complex Productive Configurations

**DOI:** 10.1177/09500170221103131

**Published:** 2022-07-04

**Authors:** Martine D’Amours, Leticia Pogliaghi, Guy Bellemare, Louise Briand, Frédéric Hanin

**Affiliations:** Université Laval, Canada; Universidad Nacional Autónoma de México, México; Université du Québec en Outaouais, Canada; Université du Québec en Outaouais, Canada; Université Laval, Canada

**Keywords:** control, employment relationship, productive configuration, risk, social labour relation

## Abstract

Increasingly, work and employment take place within network firms, value chains, and other organisational forms extending control beyond the firm’s legal boundaries. This article proposes a model rooted in sociological concepts (work organisation, control, and risk) to analyse how social relations of work and employment are structured, and how inequalities are manufactured, in these organisational forms. First, we change the level of analysis, moving from firm to productive configuration. Second, we propose the notion of *social labour relations*, to grasp the relationship between workers and any entity likely to control their conditions of work and employment. Social labour relations articulates five dimensions that could be used to compare groups of workers who are participating in the same configuration. Third, we analyse how control is exercised by which entity/entities and over which social labour relation dimension. Such an understanding is essential to provide avenues for institutional renewal: namely to reconnect control and responsibility.

## Introduction

Over the past 30 years, job instability, income insecurity and lack of social protection have affected a growing number of workers. This is explained partly by the break-up of wage labour into a wide range of non-standard statuses, and partly by the fragmentation of productive processes throughout the network firm, value chain, and other organisational forms extending control beyond the firm’s legal boundaries. These are far from marginal: the ILO estimates that more than one job in five is located within global supply chains (International Labour Organization (ILO), 2015). If domestic value chains, various forms of network enterprises, and work through digital platforms are also considered then the proportion of workers affected is greater.

In those forms, productive activities are carried out within the framework of contractual relations between legally distinct entities working to produce a single product or service. Consequently, work and employment conditions tend to deteriorate; the responsibilities that formerly belonged to a single employer are diluted, and inequalities are increasing. The literature review revealed, as did our empirical work, weaknesses in the ability of traditional models to analyse social relations of work and the structure of inequalities in those productive configurations. In particular, the classic definition of the employment relationship, characterised by subordination to the employer within the legal boundaries of the firm, is too narrow a framework to take into account these new realities.

Seeking to overcome those weaknesses, our article proposes a model rooted in sociological concepts (work organisation, control, and risk) to analyse the nature of work and employment relationships, here reconceptualised as *social labour relations* (SLR). This places a focus on the *manufacturing* of inequalities between groups of workers whose work activity and employment are embedded in organisational forms extending control beyond the firm’s legal boundaries.

This analysis has three steps:

First, changing the level of analysis. The focus shifts from the legal boundaries of the firm to the economic and sociological boundaries of the productive configuration.
Second, conceptualising work and employment, beyond the legal nature of the contract. The proposed notion of *social labour relations* (SLR) articulates five dimensions that could be applied to any form of paid work: (1) work organisation; (2) workload, hardship, schedules; (3) compensation; (4) distribution of economic risks; (5) distribution of social risks. These dimensions could be used to compare groups of workers who are participating in the same productive configuration, allowing us to identify where inequalities are located.
Third, understanding how and by whom these inequalities are generated. We expand the concept of control, here defined as any procedure or method that facilitates coordination between actors and has the effect of structuring one or more dimensions of the SLR. This makes it possible to see which control mechanism is exercised by which entity/entities over which dimension, as well as the intensity of that control.

In this article we first review research aimed at bridging the gap between inter-organisational relationships literature, and work and employment literature. Next, we acknowledge the pioneering work of Rubery and colleagues in reconceptualising the employment relationship in this context. Drawing on their work, we thereafter propose a definition and model to analyse social labour relations in organisational forms that combine deconcentrated production and fragmented control. We then provide an illustration utilising the configuration of poultry production in Quebec, Canada. The last section discusses this article’s contributions.

## Bridging the gap between inter-organisational relationships and the employment relationship

Globalisation has led to the development of analytical frameworks that focus on inter-organisational (or inter-firms) relationships. The two best known theoretical approaches to inter-firm relationships are Global Value Chains (GVC) and Global Production Networks (GPN). They have in common analysis of ‘the nexus of interconnected functions, operations and transactions through which a specific product or service is produced, distributed and consumed’ (Coe et al., 2008: 272), but they differ in their epistemological positions.

The GVC approach is rooted in transaction cost economics. It focusses on governance structures among entities forming a value chain according to three variables: (1) complexity of the knowledge and information required; (2) codifiable nature of this information; and (3) suppliers’ capabilities (Gereffi et al., 2005). The GPN approach proposes a broader relational framework that attributes importance to institutions and social norms, and emphasises power relations between multiple actors (firms, states, trade unions, NGOs). The relevant variables are value, power, and how agents and structures are embedded in particular territories (Henderson et al., 2002; Rainnie et al., 2011).

Studies of GVCs or GPNs, however, consider economic and organisational aspects rather than those related to the capital-labour relationship, such as working conditions (Nikulin et al., 2021), work organisation, and economic and social risk distribution. One of the main criticisms of these perspectives is raised by the Labour Process Theory (LPT), which claims that they do not take sufficient account of work, both as object and subject of transformation. Therefore, studies conducted within this perspective introduce the consideration of labour control in chains and networks (Baglioni, 2018; Bair and Werner, 2015). Other authors have sought to reconcile macro- and meso-perspectives of GVC/GPNs and micro-perspectives of LPT or labour market segmentation theories (Flecker and Meil, 2010; Newsome et al., 2015).

Rubery, Grimshaw, Marchington and colleagues (Grimshaw and Rubery, 2005; Marchington et al., 2005; Rubery et al., 2003) were among the first to assert the need to study the employment relationship as part of the study of inter-organisational relationships, i.e., to embed the analysis of capital-labour relationships in the analysis of capital-capital relationships. A number of authors, e.g., Dahlmann and Huws (2009), Flecker and Meil (2010), and Weil (2014), have shown that ‘increasingly, inter-firms’ relations impact more or less directly on work organisation and employment conditions’ (Flecker and Meil, 2010: 680–681). Lakhani et al. (2013) developed a model intended to predict some aspects of work and employment in subcontracting firms on the basis of the value chain configuration and the influence of institutions located in countries of the lead firm and subcontracting firm.

## A reconceptualisation of the employment relationship in complex productive configurations

Beyond demonstrating the impact of inter-organisational relationships on work or employment conditions, one might ask what efforts have been made to reconceptualise the employment relationship in this context. Here again, Rubery and colleagues were pioneers in proposing broadening the framework of analysis of employment relationships beyond the status of employee of a single employer. The diagram by Marchington et al. (2005), enables us to locate employment configurations along two axes with a twofold extension. The worker dimension (*x*-axis) includes everything from wage employment to self-employment whether the employer dimension (*y*-axis) is single or multiple.

Marchington et al. (2005: 18) noted:

“The *x*-axis depicts variations in the internalization or standardization of the employment contract and the *y*-axis the extent to which the employment contract is under the influence of a single employing organization or subject to control or influence by multiple employers”.

Thus, the *y-*axis considers all entities directly or indirectly involved in work and employment (client firms, partners, suppliers), whether or not they are legally designated as employers. The authors analyse various modalities distributed along both axes: temporary employment agencies; outsourcing; franchising; supply chains; public-private partnerships.

Our contribution draws directly on this model but adds two main contributions. First, we focus on the social relations of work and employment, beyond the contract’s legal nature, based on the sociological concepts of work organisation, control, and risk. Indeed, the nature of the contract is the outcome of a variety of actors’ struggles and institutional arrangements. Consequently, activities that are similar in terms of work organisation and risk distribution will be defined as wage labour in some countries and self-employment in others. Therefore, legal definitions must be exceeded to identify the parties involved in the relationship and to understand how they connect through social relations of power and dependency.

Our work is based on the premise that there will always be institutional mechanisms to regulate any kind of work activity in a productive system structured by relations of control over the organisation of work and by rules applying to the distribution of income and risk. Forms of regulation may be diverse (labour law, civil law, trade rules). Based on our research, we adopt a broad conception of employment relations, which we derive from the institutional codification of social relationships associated with work and activity (Bentein and Guerrero, 2008; Fouquet, 2011). From this perspective, employment goes beyond wage relations to include all the ways in which labour is made available (Dupuy and Larré, 1998). To understand the broadened figure of the worker as it extends beyond the formal wage relationship, we need to examine the various forms taken by the employment relationship using the same constitutive dimensions for each form. Dupuy and Larré (1998), for instance, suggest that all types of work performance share two dimensions in how work is organised, and how risks associated with work are distributed.

Batt and Appelbaum (2017: 76) noted that the twofold extension by Rubery and collaborators ‘leads to new types of labour market segmentation and inequality among workers’. However, and this is our second contribution, we propose that in order to understand the structure and entities responsible for inequalities in complex organisational forms it is necessary to: (1) change the unit of analysis, moving from firm to productive configuration; (2) compare workers’ conditions of work and employment, between entities and within each of the entities involved in the configuration, along the same dimensions; and (3) identify entities that, beyond legal subordination, are likely to control or structure a worker’s conditions of work and employment, by analysing which aspects of work and employment they control or structure, and with what degree of scope or intensity.

We propose to define a productive configuration as a social system in which actors divide up production activities required to provide a product or service. A productive configuration (1) represents extension of the division of labour beyond the boundaries of the firm, (2) expresses possibilities of coordinating labour beyond legal boundaries, and (3) allows dominant entities to transfer economic and social risk to entities with less power, and to their workers. As the boundaries and constituent entities of a productive configuration are not determined a priori, the researcher’s first task is to reconstruct the boundaries and entities involved, and then to analyse the social relations between them and various groups of workers.

How should we analyse social labour relations within a productive configuration, and to what dimensions should we refer? Our approach extends the concept of the wage-labour nexus (*rapport salarial*), a key concept in French regulation theory. The wage-labour nexus is ‘the configuration of mutual relations among different types of work organisation, life-styles and ways in which the labour force is reproduced’ (Boyer and Saillard, 2002 [1995]: 345). It has five analytical components:

the type of means of production; the social and technical division of labour; the ways in which workers are attracted and retained by the firm; the direct and indirect determinants of wage income; and lastly, the workers’ way of life, which is more or less closely linked with the acquisition of commodities and the use of collective services outside the market. (Boyer and Saillard, 2002 [1995]: 345)

Regulationists have studied successive forms taken by the wage-labour nexus over a long period. During the post-war period, they posited that the Fordist wage-labour nexus was uniform for industrialised countries (with national variations); they later acknowledged that a variety of ‘wage relations’ might co-exist at sector or firm level (Boyer, in Boyer and Saillard, 2002 [1995]: 77–78). However, all of these studies were limited to the classic binary wage relation, and none accounted for inter-organisational relations.

Other authors from the regulationist school suggested methods to go beyond this limitation. Lacroix and Mollard (1995) introduced the concept of the *work-social relation* to account for self-employment in the agricultural sector. This enabled them to ‘identify the specific forms taken by the capital-labour relation, and the institutions that regulate it, in a sector dominated by self-employment’ (Boyer and Saillard, 2002 [1995]: 547, authors’ translation). In her work on self-employment and hybrid forms between wage employment and self-employment, D’Amours (2013, 2014) took up and adapted the framework suggested by Lacroix and Mollard (1995), to analyse how client firms may intervene in organising work and defining rates of self-employed workers. In this project’s context, the hypothesis is that the concept of social labour relations (SLR) can be adapted to the study of various configurations of the employment relation.

SLR is defined as the relationship between workers and any entity likely to control their conditions of work and employment. The SLR concept articulates five dimensions. The first two are related to work and remaining three are related to employment.^1^ These are commonly used in sociology, but they often appear to be detached from each other:

- Work organisation, autonomy, skill level.
- Workload, hardship, schedules.
- Compensation: rules, level, extent to which it is guaranteed and predictable.
- Distribution of economic risks: job security/stability, knowledge obsolescence (employability).
- Distribution of social risks: illness, accident, parenthood, retirement.

These dimensions can be used to analyse any form of paid work (employed/self-employed; formal/informal). They allow for a comparison of groups of workers in the same productive configuration.

Our analysis of various productive configurations^2^ occurred in three stages. First, we identified the boundaries and constituent entities of a productive configuration. Second, we detected the social labour relations of groups of workers involved in that configuration, regardless of the legal or formal nature of their contract. We then compared SLR using the five analytical dimensions. Next, we studied various entities involved in *manufacturing* the SLR of various groups of workers; the relations between these entities; the ways in which they controlled one or several dimensions of various groups of workers’ SLR; and the intensity of that control. In this model, control refers to any procedure or method that facilitates coordination between actors and has the effect of structuring one or more dimensions of work and employment.

**Figure 1.**
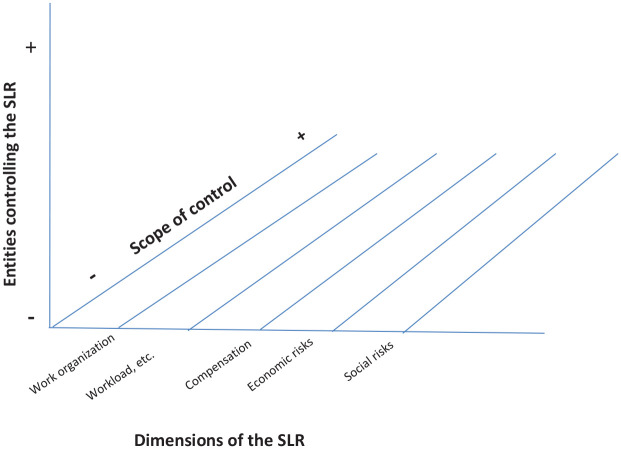
Mapping the SLR.

Our model (see Figure 1) is organised around three axes:

1. Horizontal axis (Axis 1 or *x-*axis): defines, for a given group of workers, the content of the five SLR analytical dimensions.
2. Vertical axis (Axis 2 or *y-*axis): illustrates the division of labour between *control entities*, i.e., all of the entities likely to control the SLR.
3. Diagonal axis (Axis 3 or *z*-axis): shows the degree of control (from weak control to complete domination) exercised by each entity on each of the SLR’s five dimensions.

## Poultry processing as an illustration

The model is illustrated^3^ by the case of the poultry value chain in Quebec, Canada. It is a domestic chain, protected from competition by a supply management system, which ties production levels to national demand levels and limits imports. Although national in terms of ownership of firms, the poultry chain is global because of the multinational nature of some client firms and the origin of the workforce. For example, between half and virtually all employees in chicken processing plants were born outside of Canada, and an increasing proportion are temporary foreign workers.

The institutional framework of supply management has prevented the Canadian poultry industry from achieving the vertical integration that characterises its American counterpart, where a small number of firms own hatcheries, feed mills, slaughterhouses and processing plants, and contracts with producers (Gereffi et al., 2009). In Quebec and Canada, the value chain is functionally integrated, but not integrated through ownership. Chicken producers retain considerable control over their activities and are guaranteed markets for their products at prices negotiated between the growers’ associations and processing associations. This guarantees the supply of slaughterhouses, which guarantee a minimum number of hours for their standard employees.

The design of the chain has changed significantly. Previously, production (of a limited number of minimally processed final products) was done entirely in the main plants. After a process of concentration over approximately 15 years, processing became dominated by two large lead firms that own, control or supply most of the raw material to a hierarchy of establishments. Each firm relies on several types of subcontracting i.e., specialised subcontracting for truck transport or maintenance, but also cost subcontracting, since some production activities — like deboning and further processing — have been removed from slaughterhouses and outsourced to subcontractors (e.g., Firm B) or other companies lower down the value chain.

The process of emptying henhouses has, for decades, been accomplished through occasional hiring of neighbours and family members. Driven by lead firms, it has become a formal activity, carried out by firms that specialise in chicken catching (e.g., Firm C). Their workforce is chiefly made up of temporary foreign workers (TFW) from Guatemala. Research studies (Preibisch, 2010; Robillard Dumont and Depatie-Pelletier, 2013) have highlighted that the characteristics of the TFW programme leave workers in a highly vulnerable position. Vulnerability factors include work permit restricted to one employer, for a limited period of time; employer’s power to deport a TFW to country of origin without possibility of appealing the decision; and provision of accommodation by the employer. At the time of the main survey (2016–2018), TFWs were only present in chicken-catching firms; now they constitute a new group of workers in the plants.

The phenomenon of concentration has affected not only processing, but also distribution in the poultry chain. Due to mergers and acquisitions, the market is largely controlled by five large retailers and a handful of rotisseries and fast-food chains. Through their sheer purchasing power, client firms can create competition between processors and further processors to get quantities they need at the lowest prices, and to ensure rapid delivery times and compliance with public and private standards and certifications.

The following section deals with differences in SLR experienced by labourers (unskilled workers) depending on whether they work for lead Firm A, which carries out slaughtering and first cutting; Firm B, to which A subcontracts further processing and packaging operations; or Firm C, responsible for catching and caging chickens for A. The next sections examine entities controlling each group’s SLR (Axis 2) and analyse the degree of control exercised by each entity on each SLR dimension (Axis 3).

### Multiple SLR, multiple axes of inequality

In line with this project’s central hypothesis, multiple SLR coexist in the poultry chain, depending on the position occupied in the division of labour: chicken catching, slaughter and initial cutting versus further processing. This corresponds to the concept of *segmentation*. In addition to this segmentation, distinctions are made according to the worker’s status within the firm. In Firms A and B, most workers have the status of standard employees, while a minority have the status of non-standard employees (part-time, seasonal, occasional, student). Non-standard employees do not hold permanent positions and work under less favourable conditions, even if they perform the same tasks as their standard colleagues. In the last two years, in both plants, TFWs have emerged as a new workforce component. This classification of workers according to status corresponds to the concept of *tiering*.^4^ The effect of segmentation is seen reading Table 1 from left to right, and in reading the columns from top to bottom, tiering’s effects are evident.

**Table 1.** Segmented and tiered SLR (2020).

SLR	Firm A	Firm B	Firm C
**Nature of the activity**	Slaughtering and first cutting	Further processing	Chicken catching
**Union/non-union**	Unionised	Unionised	Non-unionised
**Work organisation**			
Standard employees	Job holders	Multiple tasks within a job class; greater versatility than in A	Majority of TFWs, except for the team leader and assistant
Non-standard employees	Not job holders	Assigned as needed for replacement	
TFW	As required, especially to clean equipment	Assigned as needed	
**Workload, hardship, schedules**			
Standard employees	Day or night shift Shift premium 12 paid holidays	Day or night shift Shift premium 10 paid holidays	Night shifts, no premium 8 paid statutory holidays
Non-standard employees	Can change shift depending on replacement; same premium and paid holidays	Can change shift depending on replacement; shift premium but no paid holidays	
TFW	Mostly on the night shift; same premium and paid holidays		
**Compensation (Canadian dollars)**			
Standard employees	$16.57 at entry $18.64 after 1 year $20.71 after 2 years	$14.54–$15.73 at entry $15.26–$16.51 after 700 hours $16.02–$17.34 after 4,000 hours	Payment by the hour or by the load Legal minimum wage, sometimes less
Non-standard employees	Same except for students	Same	
TFW	$16.57	$14.54–$15.73; 15.26–$16.51 after 700 hours	
**Economic risk**			
Standard employees	Minimum guaranteed compensation for 35 hours per week (after 3 years)	No guaranteed working hours or compensation	No guaranteed working hours or compensation
Non-standard employees	No guaranteed working hours or compensation	Same	**___**
TFW	35 hours in principle. In practice, no guaranteed hours The main risk: breach of the employment contract and repatriation to the country of origin		
**Social risk**			
Standard employees	After 700 hours: group insurance, dental care	Group insurance (less favourable than A) Dental care after 700 hours	Separate, less expensive insurance scheme
Non-standard employees	After 700 hours, entitled to benefits, except sick leave and dental care Students not eligible	Not entitled to benefits or holidays; compensatory bonus of 8% (10% after 3 years)	___
TFW	Contribute to public programmes but unlikely to receive benefits (except for medical care paid for by public scheme) Separate, less expensive insurance plan		

SLR, social labour relations; TFW, temporary foreign worker.

With regard to organisation and workload, employees of the lead Firm A and those of its subcontractors B and C have three things in common: (1) they have very little autonomy in doing their work, (2) except for the evisceration activity (exclusive to A), which requires six weeks of training, no particular skill is required; and (3) the workload has intensified, and workers face difficult health and safety conditions. The labour shortage that has plagued the sector for years is largely due to these difficult conditions. The difference between them lies in functional and temporal flexibility that is demanded of Firms B and C’s employees.

The effect of segmentation is most visible on the employment dimensions. Table 1 shows that hourly pay decreases as one moves from A to B to C. Firm A’s standard employees are the only ones protected against risk of economic fluctuation because after three years of service they are guaranteed 35 hours of weekly pay. Firm B’s workers, by contrast, play the part of an adjustment variable: they have no guaranteed number of hours, but are required to do overtime when large orders are received.

Chicken catchers are not guaranteed work hours, even though federal government requires that a 35-hour work week be provided. Their main economic risk is breaking the employment relationship, which would mean repatriation to their country of origin. This explains why, even though their employers must in theory respect federal and provincial laws, their isolation and lack of information, their dependence on an income that is higher than that of their country of origin and their fear of breaking the employment link (or not being called back the following year) makes abuse possible. For example, although Quebec’s government mandates the current minimum wage for TFWs in the poultry sector (C$13.10/hour as of 1 May 2020), their pay may fall below minimum wage because the time it takes to travel between two farms is not always compensated.

For Firm A’s standard workers, social risk is collectively shared: they are covered by public social security programmes (employment insurance, compensation for workplace injuries, pension benefits), and the employer contributes to the cost of other, complementary social safeguards (group health insurance). Firm B’s employees are also covered by public programmes, but the lack of a guaranteed number of working hours and their low hourly wage mean that benefits, when paid, are small. They have a group insurance plan, but its terms are not as good as those granted to A’s workers. This is also true for C’s employees. The SLR is more favourable for A’s than for C’s employees while B’s employees occupy a middle position as seen in Table 1, even though the further processing they carry out creates greater added value for the lead firm than the slaughtering in A.

SLR also vary within each segment, according to the hierarchy of employment status inside each firm. In Firms A and B, standard employees fare better than their non-standard counterparts. Non-standard workers are called in when there is work, but they are the first to be let go when there is no work. Their revenue, therefore, is unstable. They are required to work on variable tasks and schedules. Part of the social risk is mutualised, and the other part is assumed by workers. They contribute to various public programmes but are less likely to fulfil the conditions required to receive benefits, and if they do, the benefits will be basic. Moreover, coverage applies only for the contract’s duration.

Both A and B have recently hired TFWs, who share the vulnerability of C’s workers, but with more favourable pay and social protection provided by A and B’s collective agreements. However, TFWs, who are most often hired on nine- or ten-month contracts, will not easily obtain pay increases attached to one- or two-year terms. TFWs must contribute to several public benefit programmes (employment insurance, parental leave, pensions), but they rarely receive benefits.

### Control without responsibility

Two questions must be posed: what are the entities that structure these differential working conditions and employment terms?; and which dimensions of SLR do they act on and how intensely? To find answers it is necessary to understand power relations between lead Firm A and its subcontractors. Firm B’s relation with Firm A, its only client, is of total dependence. Firm A negotiates contracts with end clients, owns and installs equipment used by B, provides B with raw material and certifies product quality. Research and development, and choosing products and recipes, are also A’s prerogative. Due to its subcontractor, Firm A can increase its capacity as a supplier to client firms.

Firm C is one of the few chicken-catching businesses subcontracting for A, which dictates, directly or indirectly, a number of conditions. In particular, it is rarely possible for chicken-catching businesses to negotiate prices, because the environment is so competitive. In both cases, Firm A, whose name appears on the products, is responsible for ensuring compliance with standards. This is why it exercises control over subcontractors. This also explains why the role of the legal employer is more limited in subcontractors than in the lead firm (see Table 2).

**Table 2.** Entities involved in each dimension of SLR of each group of workers. .

Entities involved in each dimension of SLR	Firm A	Firm B	Firm C
**Work organisation**	**Legal employer** - Hiring, supervision, training, discipline - Choice of products and work processes - Choice of technology and equipment - Control over work organisation	**Legal employer** Hiring, supervision, training, discipline	**Legal employer** - Hiring, supervision, training, discipline - Finding accommodation
		**Lead Firm A** - Choice of products and work processes - Choice of technology and equipment - Control over work organisation - Quality control through audits	**Lead Firm A** - Catching techniques - Training - Load quality control + audits
	**End clients** - Standards applying to prices, quantities, time frames, etc. - Quality control through audits	**End clients** - Standards applying to prices, quantities, time frames, etc., passed on by Firm A - Quality control through audits	
**Workload, hardship, schedules**	**Legal employer** - Work schedules - Bonuses and days off - Occupational health and safety (OHS) conditions	**Legal employer** - Work schedules (to some extent) - Bonuses and days off (within the limits of the contract negotiated with Firm A) - OHS conditions (within the limits of the work organisation and equipment chosen by Firm A)	**Legal employer** - Days off (minimum legal) - OHS conditions (in part)
		**Lead Firm A** - Variation in the number of working hours - Work schedules (to some extent) - OHS conditions (through choice of technology and equipment installation)	**Lead Firm A** - Work schedules and routes
			**Poultry producers** Hygiene and safety conditions in henhouses
	**End clients** Standards applying to quantities and delivery times	**End clients** Standards applying to quantities and delivery times, passed on by Firm A	
**Compensation**	**Legal employer** Determines wages (through negotiation with Firm A’s labour union)	**Legal employer** Determines wages (through negotiation with Firm B’s labour union), within limits of contract signed with Firm A	**Legal employer** Determines wages (without negotiations) within limits of contract signed with Firm A
		**Lead Firm A** Establishes the rate and method of payment (C$ per kg); defines the limits of compensation that the legal employer can provide	
	**End clients:** Create competition between processing firms to get the lowest possible price		
**Economic risk**	**Legal employer** Guaranteed minimum compensation	**Legal employer** No guaranteed hours or minimum compensation	**Legal employer** May terminate employment relationship, which means repatriation to country of origin
			**Lead Firm A** Can require TFW to stop working on its loads
		**Lead Firm A** Protects its own standard worker and passes on to subcontractors risk that orders from customers (and working hours) will fluctuate	
	**End clients:** Fluctuating orders		
			**Federal government** Establishes characteristics of TFW programme; closed permit that empowers employer
**Social risk**	**Legal employer** Private group insurance plan	**Legal employer** Less generous private insurance plan	**Legal employer** Minimal private insurance plan
		**A** Volume contract with Firm A defines limits of protection that legal employer can provide	
	**Government** Coverage through public programmes	**Government** Coverage through public programmes, but lower level of coverage because working hours fluctuate and hourly wage is low	**Government** Public programmes do not apply in practice

Note: OHS, occupational health and safety; SLR, social labour relations; TFW, temporary foreign worker.

In the case of Firm B, the legal employer controls hiring, provides (limited) training and compensation and ensures supervision and discipline. However, B’s employees do their job within a work organisation (technology, equipment, methods) determined by A, with a possible impact on occupational health and safety. Firm B’s workers have no guaranteed number of hours, mainly because A gives its own employees hours (including overtime) before it uses B workers.

Regarding C’s employees, A establishes chicken-catching schedules, tying them to the slaughter schedules. It influences training of team leaders, to teach them working methods that respect government and private regulations concerning animal welfare. Firm A also evaluates quality of work performed by the subcontractor in various ways: inspection when the load arrives and audits to verify that standards have been respected. In extreme cases, slaughtering firms can require that their subcontractors stop assigning a particular worker to their loads, which may result in the worker’s dismissal. For both B and C, the salary and benefits that the legal employer is able to offer employees depends on the terms of the contract negotiated with A.

The control by the end users applies at two levels: the dimensions of the SLR and the configuration of the chain. Various elements of the SLR (derived from product specifications, delivery times, quality assessment) are imposed by the client firms, and are agreed upon in a commercial contract with A, which passes them on to its subcontractors. The lower hourly wage of B and C’s employees is a condition enabling A to obtain contracts with client firms. The contracts are signed because lead Firm A is able to supply products that meet quality requirements at the lowest price, and this low price is achieved by compressing the cost of labour in peripheral plants and creating tiered employment status in main plants. To face the competition implemented by client firms, lead processing companies have adapted their strategies. This motivated, for example, the decision to have some further processing, deboning, and packaging done in plants where working and employment conditions are less favourable or the decision to use the TFW programme.

In the case of Firm C, poultry producers and the federal government (which is responsible for the TFW programme) are control entities. Poultry producers determine some health and safety conditions under which catchers work. These conditions include, for instance, access to toilets, the presence or absence of balconies on the upper floors of henhouses, and poor ventilation. Finally, because of their exceptional status, TFWs are far more dependent on their employers than Canadian citizens or permanent residents. While labour laws apply in theory, they are largely ineffective in practice, particularly because workers do not dare to complain in case of an accident at work or if their rights are not respected.

To summarise, in the poultry chain, the lead firm subcontracts production activities that were once part of its ‘core business’ in order to comply with client firms’ requirements in terms of quality, time, and especially prices. The unequal relationship between A and B, added to competition between lead firms to obtain contracts from client firms, creates the inferior working and employment conditions experienced by people working for the subcontracting firm. This relationship also reduces the ability of both groups of workers to act collectively, since A uses its ability to displace production segments (by keeping them in-house or externalising them) to limit demands of its own employees and their unions and to make sure that low costs will continue to be required from the subcontractors.

In each dimension studied, the SLR of Firms B and C’s employees is much less favourable than that of Firm A’s employees. Inside each firm, some tiered employment statuses are associated with worse conditions (Axis 1). In addition, the SLR of B and C’s employees are controlled by three entities with unequal powers: their legal employer, lead Firm A, and client firms (Axis 2). Dominant entities extend their control beyond their borders to structure SLR of employees of other entities, while shifting responsibility for protection that lies with the legal employer onto entities with fewest power resources. Each entity acts with more or less intensity on each SLR dimension (Axis 3). Looking at the three elements of managerial rights — managing business, managing production and managing labour^5^ — it is clear that B exercises only the right to manage labour, and its role is essentially limited to providing the workforce. The situation is different for standard employees of slaughterhouses: except for the role of client firms, which is all-pervasive in the agri-food processing sector, the legal employer has almost complete control over them.

## Discussion

In order to overcome the weaknesses of the traditional models of analysis of work and employment, there are three main contributions made by this model:

1. The first contribution is the change in the level of analysis, moving from the legal boundaries of the firm to the economic and sociological boundaries of the productive configuration. Since it goes beyond the worker’s status, the nature of the contract and the physical and legal boundaries of the entities participating in the configuration, this allows several types of productive configurations to be analysed, such as value chain (poultry processing), network firm (childcare services), project team (IT company services) or platform work (Uber).^6^ Although other authors have situated their analysis at the *meso* level of network enterprises or value chains, we propose a definition that can be applied to various organisational forms that extend control over work beyond the firm’s legal boundaries. A productive configuration (1) represents the extension of the division of labour beyond the firm boundaries, (2) expresses the possibilities of coordinating labour beyond legal boundaries, and (3) allows dominant entities to transfer economic and social risk to entities with less power, and to their workers. The result is that workers who perform similar tasks are given different conditions of employment and work, depending on the position of their employer in the configuration and on their own position inside their firm.
2. We propose to reconceptualise the employment relationship as *social labour relation* (SLR). SLR is defined as the relationship between workers and any entity likely to control their conditions of work and employment. The concept of SLR and its five constituent dimensions make it possible to compare groups of workers participating in a given configuration along the same analytical dimensions. Analysing disparities in work and employment conditions at the level of the productive configuration is more meaningful than a comparison based on national laws and international conventions, which uses the firm or establishment as a framework (D’Amours, 2022). On the one hand, it makes it possible to analyse multiple axes of structuring inequalities (segmentation; tiering) and to cross-reference them with other social relations of domination (e.g., immigrant workers). On the other hand, by extending the analysis of such disparities beyond the legal boundaries of the firm, researchers are able to question justification for these inequalities. For example, although workers in Firms A and B do not do exactly the same work, they do perform very similar and probably equivalent tasks, which do not justify such disparities.
3. The proposed model also makes it possible to ‘enter the black box’ of the control of work and employment by entities other than the legal employer in complex productive configurations. In this context, we propose an expanded definition of control. Control involves any procedure or method that facilitates coordination between actors and has the effect of structuring one or more dimensions of work and employment. This definition enables the observer to understand the type of control being exercised by which entity or entities over what dimensions of the SLR. Such an understanding is essential to provide avenues for institutional renewal: namely to reconnect control and responsibility. Indeed, in the productive configurations studied, there is a decoupling of the exchange of control and responsibility that characterised the standard employment relationship (Supiot, 1996), which allows for a decommodification of work. If responsibility is to be attributed to the entities that exercise control, it is essential to identify which dimensions of the SLR are influenced by which entities. As noted by Prassl and Risak (2016) the exercise by one or more entities of one or more of the functions of the employer should trigger responsibility in the applicable area of law.

Thus, this reconceptualisation makes it possible to understand the SLR of a given group of workers, at a given time, by combining its analytical dimensions in a single picture that portrays the control exercised by various entities. The synchronic perspective is complemented by a diachronic, socio-historical perspective. This allows the researcher to understand how the SLR was established and how it is changing or might change over time. This means that the collective action processes of workers and control entities must be considered. For example, a trend identified in our research is that, within the slaughtering and further processing segments, traditional non-standard employees are disappearing, giving way to TFWs who offer unparalleled flexibility to control entities. Trade unions, whose members are also affected by labour shortages, have agreed to the presence of TFWs but are concerned about the effect on their bargaining power of a recent increase (from 10 to 30%) in the proportion of foreign workers in vulnerable situations.

Although the proposed concept of social labour relation is broad, its operationalisation in five dimensions, and the model developed in this article, make it sufficiently robust to support a rigorous, non-deterministic analysis of contemporary employment relations. In contrast to Lakhani et al.’s (2013) thesis, who suggests that different value chain configurations will lead to different employment relations strategies and outcomes, we argue that SLR cannot be predicted by the configuration of a chain or network since it involves a variety of entities — client, subcontractor, labour union, regulatory agency, etc., whose identity and action cannot be known a priori. A determinist perspective is precluded by the combination of entities involved in each dimension of SLR and the variable scope of their action, and as well as the role played by public and private regulation of labour or the product/service.

## Notes

1. Work is related to how work is organised and degree of autonomy it gives the worker. Employment is related to contractual conditions under which work activity takes place; these include work contract’s degree of security, compensation, and social benefits.

2. This reconceptualisation is derived from a research project, entitled *Les nouvelles configurations de la relation d’emploi et leurs impacts sur le travail, l’emploi et l’action collective*, whose general objective was to understand, from a historical and comparative perspective, the operation of organisational forms that combine deconcentrated production and fragmented control, and the impact of these forms on work, employment, and workers’ collective action. The aim was (1) to test the hypothesis that these organisational forms result in the coexistence of patterns of work and employment with distinct characteristics (Flecker and Meil, 2010; Lakhani et al., 2013); and (2) to analyse how and by which actors these inequalities are produced, in particular contexts and institutional environments. The research strategy was based on a multiple-case study design (Eisenhardt, 1989; Yin, 1989). Four sectors, distinguished by the nature of the product/service, the strategy of the lead firm and the extent of workers’ skill levels, were targeted for analysis: passenger transportation (taxis); agri-food production and processing; information and communication technology business services; childcare services. In each sector, one or more synchronic and diachronic case studies were carried out for one or more lead companies and their subcontractors, agency workers, and self-employed workers. Each case study proceeded through the three stages identified above. Each case study draws on a variety of sources to enable triangulation: documentary sources (press articles, ‘grey’ literature); analysis of collective agreements; semi-structured interviews with key informants to reconstruct the productive configuration from a historical perspective; and interviews with representatives of employers, trade unions and other associations; and various subgroups of workers. For each case, data were coded into a single database, using NVivo 10 software. A thematic synthesis of each case was submitted to key informants and participants as an additional validation step. Next came the inter-case analysis from which we derived the proposed model.

3. Research was conducted between 2016 and 2018, updated in 2021. Additional information is provided in two research reports: D’Amours and Belzile (2019); D’Amours and Deshaies (2019).

4. Hammer and Riisgaard (2015: 89–90) conclude that, in chains or networks, we find not only divisions between workers carrying out different steps of the labour process (described by the concept of ‘segmentation’) but also complex, overlapping divisions between groups of workers who are carrying out the same tasks (known as ‘tiering’).

5. According to the analytical model by Vallée and Bourgault (2018: 93–94).

6. The model was used to analyse all cases studied in the project.
